# Supplementary material for: A lateral hypothalamic region supporting diverse visual processing and modulation of visually-guided behaviour
Source: Nat Commun. 2025 Nov 11;16:9917. doi: 10.1038/s41467-025-64864-3 (PMC12606360; doi:10.1038/s41467-025-64864-3)
Supplement: Supplementary file 1 — Supplementary Information [file 41467_2025_64864_MOESM1_ESM.pdf]

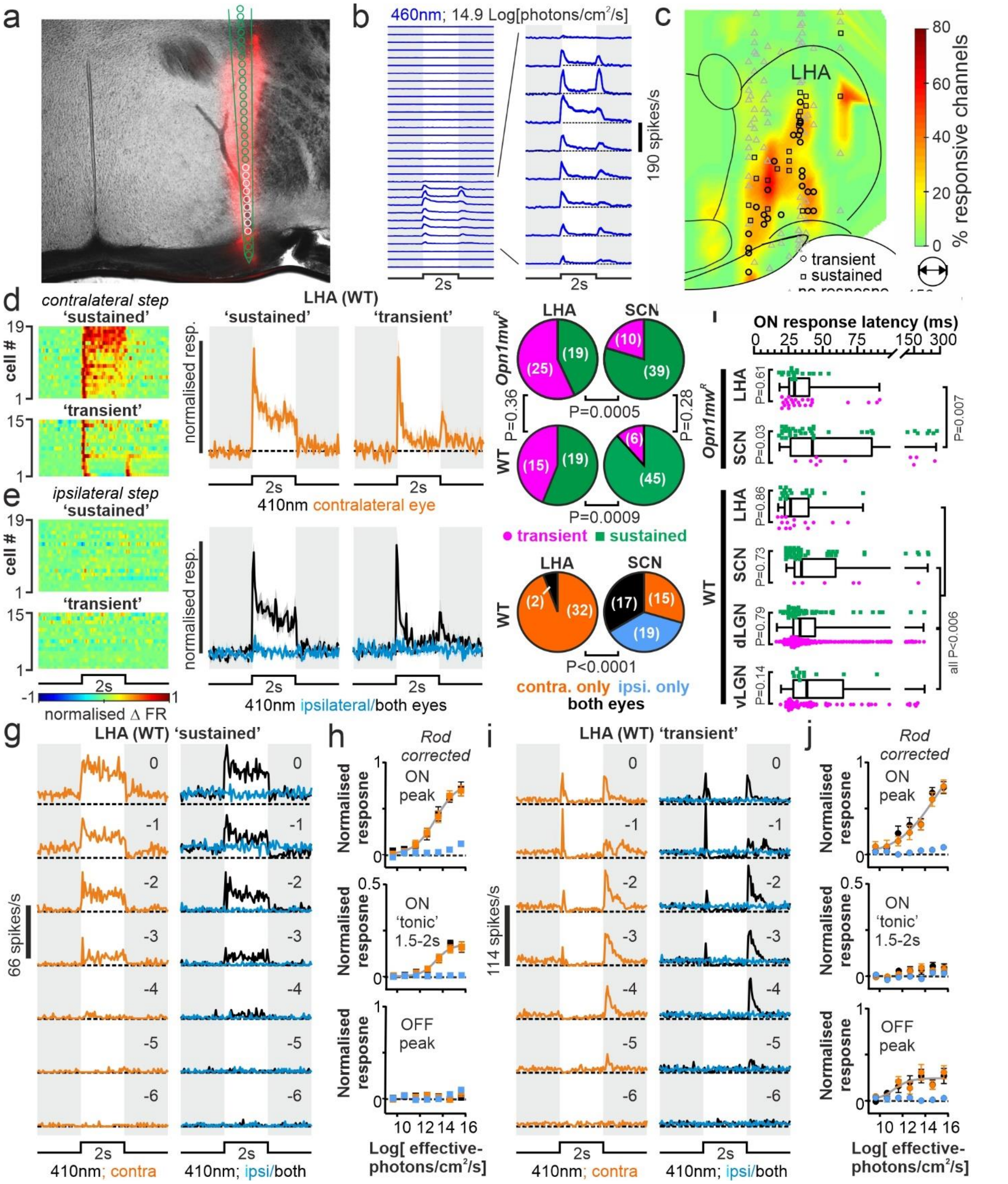

Fig S1. Contralateral light-evoked activity across mouse lateral hypothalamic neurons. (**a,b**) Additional example *Opn1mw^R* recording, showing probe track in the LHA (a) and corresponding multiunit activity (MUA) following 2s contralateral light steps (conventions as in Fig 1a,b). (**c**) Proportions of channels with significant MUA responses (n=2176 electrode sites from 68 probe placements in 20 *Opn1mw^R*mice, 150µm diameter binning); superimposed symbols indicate locations of neurons (isolated from 18 recordings/18 mice) (n=19,25 and 104 sustained, transient or unresponsive cells respectively). (**d**) Left: normalised responses of sustained and transient wildtype (WT) mouse LHA neurons, Middle: mean±SEM responses across sustained and transient cells (n=19 and n=15 respectively from 18 mice) following 2s contralateral light steps (410nm; 15.8 log photons/cm^2/s). Right: proportions of sustained vs. transient cells in LHA and SCN recordings from *Opn1mw^R* and WT mice (analysed by Fisher's exact tests; WT SCN data from[28]). (**e**) Left:

normalised response to ipsilateral light steps for sustained and transient WT mouse LHA neurons (same populations as **d**), right: mean±SEM population responses following 2s light steps applied to ipsilateral or both eyes. Pie charts show proportions of monocular or binocular cells in the LHA vs. SCN of WT mice (from[28], as above), compared by χ^2-test. (**f**) Response latencies for *Opn1mw^R* and WT, sustained and transient, neurons (box and whisker plot shows pooled distribution for each region, box=quartiles 2-3, centre=median, whiskers=min-max; WT SCN data from[28], dorsal and ventral lateral geniculate d/vLGN, data from[42]). Latencies compared between sustained and transient cells within regions by Mann-Whitney U-tests, pooled population data compared across regions by Kruskall Wallace test ($KW_{6,656}$=28.31, P<0.0001) followed by Dunn's post-tests. (**g,i**) Mean firing of representative sustained (**f**) and transient (**h**), WT, LHA neurons following 410nm light steps applied to contralateral (left), ipsilateral or both eyes (right) across 7 log-spaced irradiances. (**h**,**j**) Mean±SEM normalised responses of sustained (**g**, n=19) and transient (**i**, n=15) WT LHA to 410nm light steps as a function of rod-effective irradiance. Data fit by 4-parameter sigmoid curves and compared between contralateral-only and both eye stimulation by F-test (**h**, ON peak: $F_{2,261}$=0.003, P=0.99, 'tonic': $F_{2,261}$=0.27, P=0.76; **j**, ON peak: $F_{2,205}$=0.49, P=0.61, 'tonic': $F_{2,205}$=0.03, P=0.97).

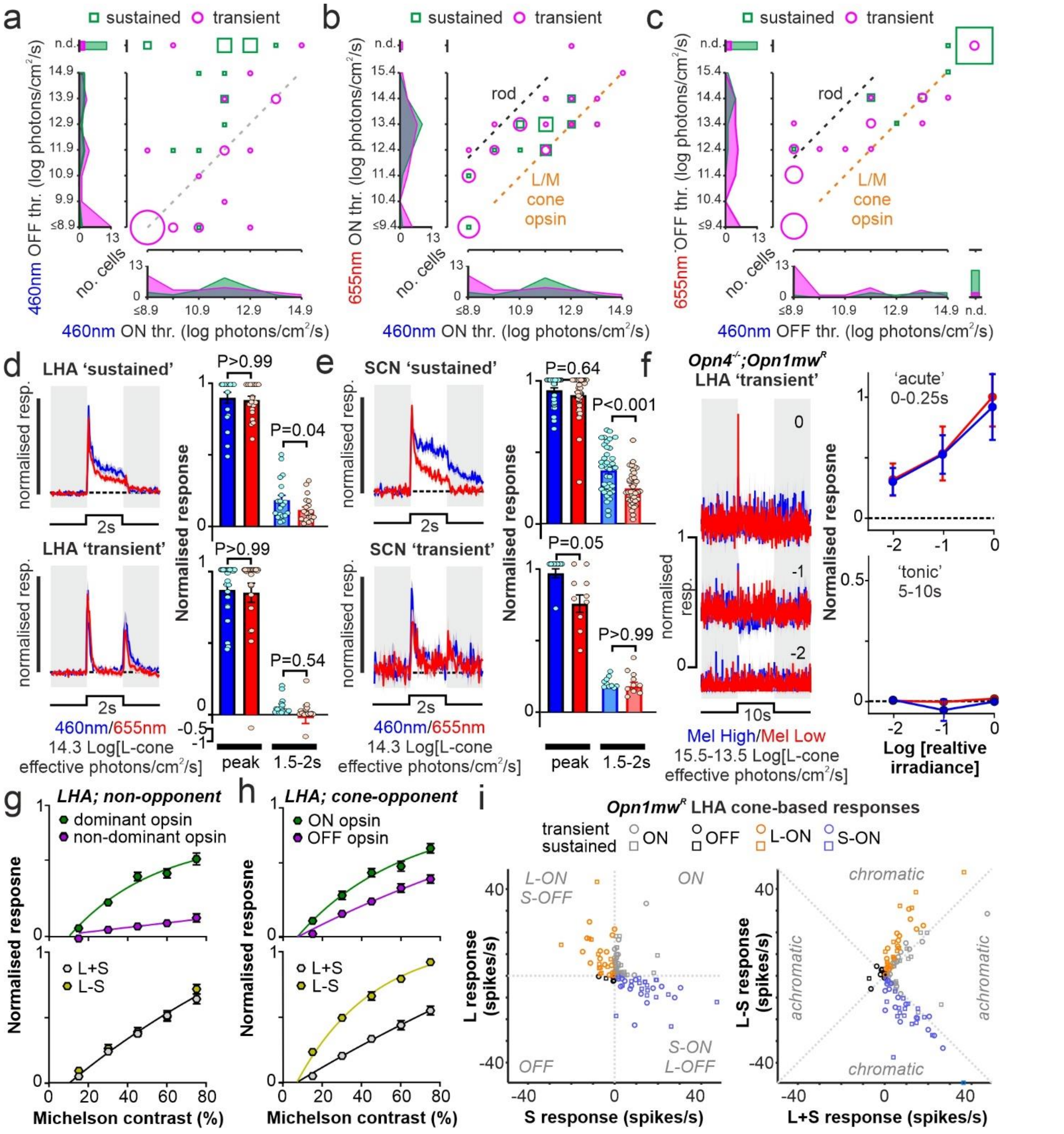

Fig S2. Rod, cone and melanopsin input to subsets of lateral hypothalamic neurons. (**a**) Thresholds for ON and OFF responses to 460nm light across sustained (n=19) and transient (n=25) LHA neurons from *Opn1mw*^{R} mice (dataset in Fig 1); symbol sizes proportional to number of cells, n.d.=no detectable response. (**b**,**c**) Thresholds for ON (**b**) and OFF (**c**) excitatory responses to 460 vs. 655nm light steps (conventions as in **a**). Dotted lines represent expected relative sensitivity to 460 vs. 655nm for responses driven by rods and L-cone opsin. (**d, e**) Responses of LHA (**d**) and SCN/peri-SCN (**e**) *Opn1mw*^{R} neurons, classified as sustained (n=19 and n=39 respectively) or transient (n=25 and n=10 respectively), to 2s L-cone opsin isoluminant 655nm and 460nm light (both 14.3 log L-cone effective photons/cm^2/s). Left: normalised mean±SEM peristimulus histograms, Right: normalised Mean±SEM response during ON peak and last 500ms of the 2s light step; data analysed by paired t-tests with Bonferroni correction (**d** upper, peak: t=0.0, tonic: t=2.3; df=18; **d** lower, peak: t=0.0, tonic: t=0.6; df=24; **e** upper, peak: t=0.5, tonic: t=7.7; df=38; **e** lower, peak: t=2.2, tonic: t=0.0; df=9). (**f**) Left: Mean±SEM normalised responses of *Opn4*$^{-/-}$*;Opn1mw*^{R} transient cells

(n=16) to 10s Mel. High and Mel. Low light steps across 3 log-spaced irradiances. Right: Mean±SEM normalised change in firing during ON peak and last 5s of the 10s light steps respectively. Data analysed by RM 2-way ANOVA (**f** upper: Irrad.-$F_{2,30}$= 9.1,P<0.0001, Stim.-$F_{1\ 15}$=0.3, P=0.58, Irrad.XStim.-$F_{2,30}$=0.1, P=0.95; **f** lower: Irrad.-$F_{2,30}$= 1.3,P=0.30, Stim.-$F_{1,15}$=0.7, P=0.46, Irrad.XStim.-$F_{2,30}$= 0.40, P=0.67). (**g,h**) Mean±SEM normalised contrast response relationships for single (upper) and dual (lower) cone opsin modulating stimuli for non-opponent (**g**) and opponent (**h**) *Opn1mw*^{R} LHA cells (n=63 and 60 respectively; corresponding to populations in Fig 2j-o). Responses to single-cone stimuli are respectively sorted according to the opsin that produced the greatest response (**g**, non-opponent cells) or the opsin that drove ON responses (**h**, opponent cells. (**i**) Mean response amplitudes to S_{Only} vs. L_{Only} stimuli (left) and L+S vs. L-S stimuli (right), showing cell classification for units underlying analysis in Fig 2j-o.

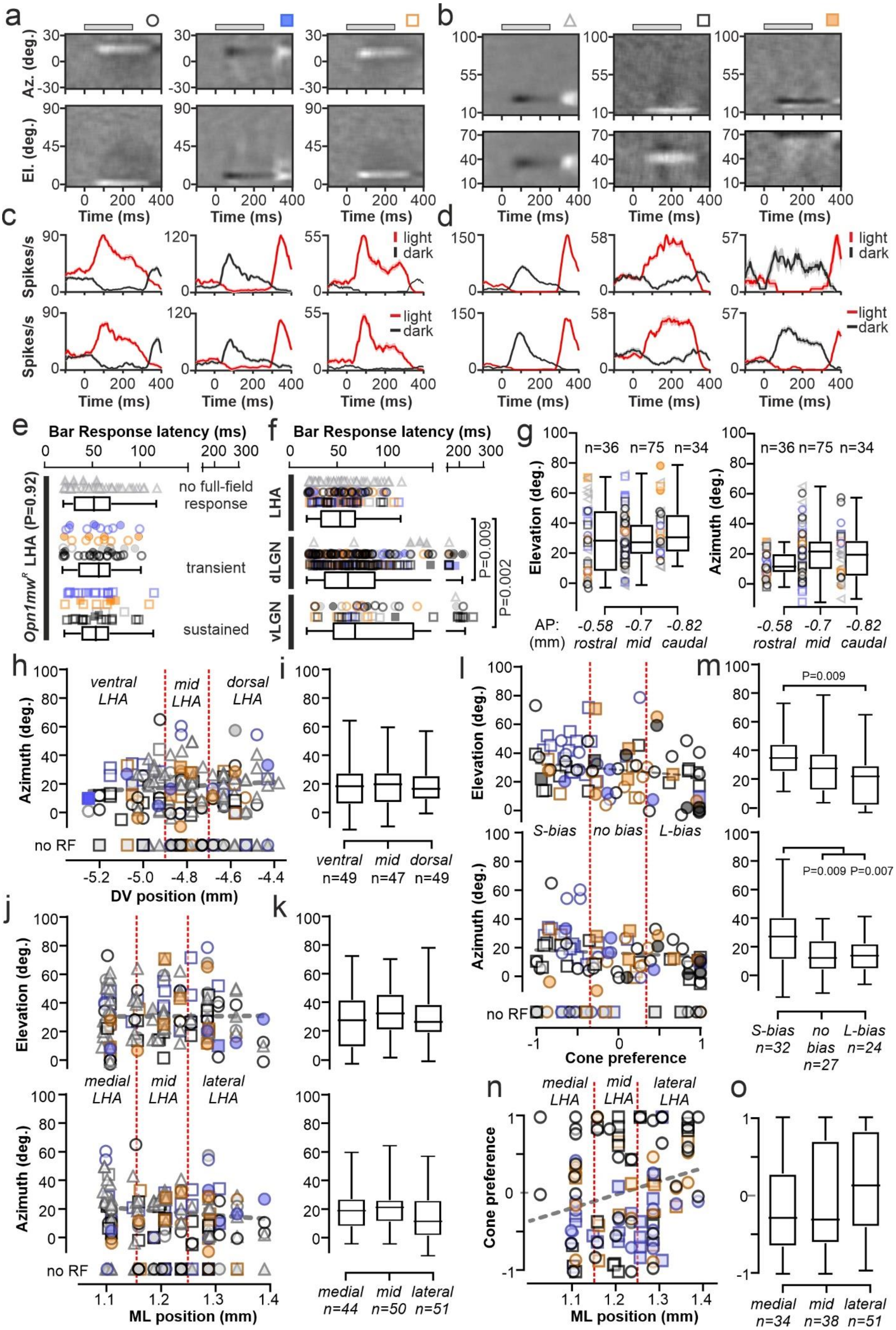

a
b
c
d
e
f
g
h
i
j
k
l
m
n
o
Az. (deg.)
El. (deg.)
Time (ms)
Spikes/s
light
dark
Bar Response latency (ms)
Opn1mw^R LHA (P=0.92)
no full-field response
transient
sustained
LHA
dLGN
vLGN
P=0.009
P=0.002
Elevation (deg.)
Azimuth (deg.)
n=36
n=75
n=34
AP: (mm)
-0.58 rostral
-0.7 mid
-0.82 caudal
ventral LHA
mid LHA
dorsal LHA
no RF
DV position (mm)
ventral n=49
mid n=47
dorsal n=49
S-bias
no bias
L-bias
Cone preference
P=0.009
P=0.007
S-bias n=32
no bias n=27
L-bias n=24
medial LHA
mid LHA
lateral LHA
ML position (mm)
medial n=44
mid n=50
lateral n=51
medial n=34
mid n=38
lateral n=51

Fig S3. Anatomical variation in lateral hypothalamic neurons receptive field properties. (**a**,**b**) Spatiotemporal response profiles for additional representative neurons with RFs detected when the monitor was positioned in frontal (**a**) or lateral visual space (**b**), conventions as in Fig 3d,e. (**c**,**d**) Mean±SEM responses to vertical (upper) or horizontal (lower) bars within the RF centre for neurons in **a** and **b**. (**e,f**) Onset latencies for *Opn1mw*^{R} responses to optimal bars; Plots (box=quartiles 2-3, centre=median, whiskers=min-max) show pooled distribution for LHA cells with transient, sustained or no response to full field stimuli (**e**; n=51,42,52 respectively) or for all responsive cells in the LHA vs. dLGN and vLGN cells tested with identical stimuli[42] (**f**; n=145, 211, 42 respectively). Data compared by Kruskall-Wallace tests followed by Dunn's post-tests (**e**: $KW_{3,145}$=0.17, P=0.92; **f**: $KW_{3,398}$=13.7, P=0.001). (**g**) Relationship between RF position and recording location across rostral-caudal LHA. (**h,j**) Relationship between RF position on azimuth (**h** and **j**, lower) or elevation plane (**j**, upper) vs. dorsal-ventral (**h**) and medial-lateral (**j**) location in the LHA for cell populations shown in Fig 3. (**i,k**) RF centre positions for cells located in ventral-dorsal (**i**) or medial-lateral (**k**) portions of the LHA (as denoted by dotted lines in **h** and **j**). (**l**) Relationship between RF position on elevation (upper) or azimuth plane (lower) vs. cone preference (n=63/117 and n=20/28 cells with ON and OFF RFs contributing to **g-j** and Fig 3c and h-j). (**m**) Distribution of RF positions on elevation (upper) or azimuth plane (lower) for S-cone biased, unbiased and L-cone biased cells (as indicated by dotted lines in **l**). (**n**) Relationship between cone preference and medial-lateral LHA position for n=124 cells responding to full-field cone-isolating stimuli (from 9 *Opn1mw*^{R} mice). (**o**) Distribution of cone preferences for cells located in medial-lateral LHA (as indicated by dotted lines in **m**). Throughout, colour coding as per conventions in Fig 3. Data in **g-o** analysed by one-way ANOVAs (**g** left: $F_{2,142}$=1.08, P=0.34; **g** right: $F_{2,142}$=2.99, P=0.054; **i:** $F_{2,\ 142}$=0.16, P=0.85; **k** upper: $F_{2,142}$=1.55, P=0.22; **k** lower: $F_{2,142}$=2.77, P=0.07, **m** upper: $F_{2,\ 80}$=4.66, P=0.01; **m** lower: $F_{2,80}$=6.58, P=0.002, **o**: $F_{2,120}$=2.98, P=0.54), with Tukey's post-tests.

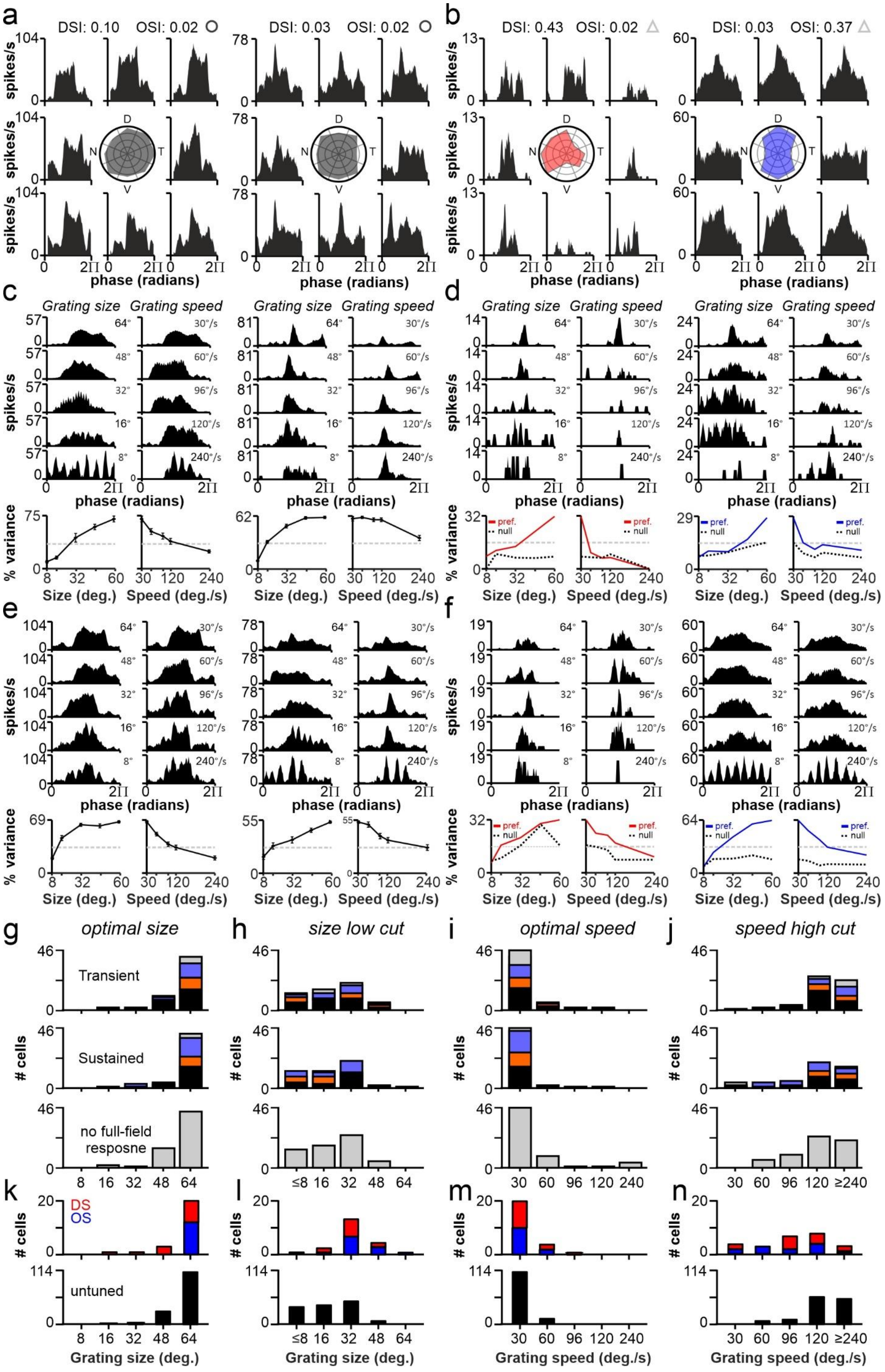

a
DSI: 0.10 OSI: 0.02
DSI: 0.03 OSI: 0.02
b
DSI: 0.43 OSI: 0.02
DSI: 0.03 OSI: 0.37
spikes/s
phase (radians)
c
d
Grating size
Grating speed
% variance
Size (deg.)
Speed (deg./s)
pref.
null
e
f
g
optimal size
h
size low cut
i
optimal speed
j
speed high cut
Transient
Sustained
no full-field resposne
cells
k
l
m
n
DS
OS
untuned
Grating size (deg.)
Grating speed (deg./s)

Fig. S4. Spatiotemporal tuning across lateral hypothalamic neurons. (**a**, **b**) Peristimulus histograms showing firing of additional representative untuned (**a**) and DS or OS (**b**) cells to gratings of optimal spatial and temporal frequency across the 8 tested direction of motion. Central rose plot shows normalised response amplitude as a function of grating direction. (**c-f**) Tuning properties for representative cells in Fig 4a,b and Fig S4a,b (c-f respectively); top panels show responses of as a function of grating size at optimal temporal frequency/angle (left) or as a function of grating speed at optimal spatial frequency/angle (right). Lower panels show response robustness (% variance accounted for by stimulus) as a function of grating size and speed; for **c** and **e** values are averaged across all tested direction, for **d** and **f** values plotted separately for preferred vs null direction/orientation. (**g-j**) Respectively, distributions of grating size that produced optimal responses (**g**), minimal grating size that evoked significant responses (**h**), grating speed that produced optimal responses (**i**) and fastest speed that evoked significant responses (**j**) across cells classified as transient (top),sustained (mid) or those that lacked full field responses (colour coding on bars indicates cells classification as non-opponent, L-ON, S-ON or no response to cone-directed stimuli). (**k-n**) Distributions of tuning properties (as in **g-j**) as a function of cells' classification as DS/OS or untuned. Data in **g** to **n** compared by χ^2-tests (**g**: χ^2=6.1, d.f.=6, P=0.43; **h**: χ^2=0.8, d.f.=6, P=0.99; **i**: χ^2=6.3, d.f.=6, P=0.40; **j**: χ^2=6.8, d.f.=6, P=0.34; **k**: χ^2=0.9, d.f.=3, P=0.83; **l**: χ^2=18.67, d.f.=3, P=0.0003; **m**: χ^2=2.6, d.f.=3, P=0.45; **n**: χ^2=21.0, d.f.=3, P=0.0001)

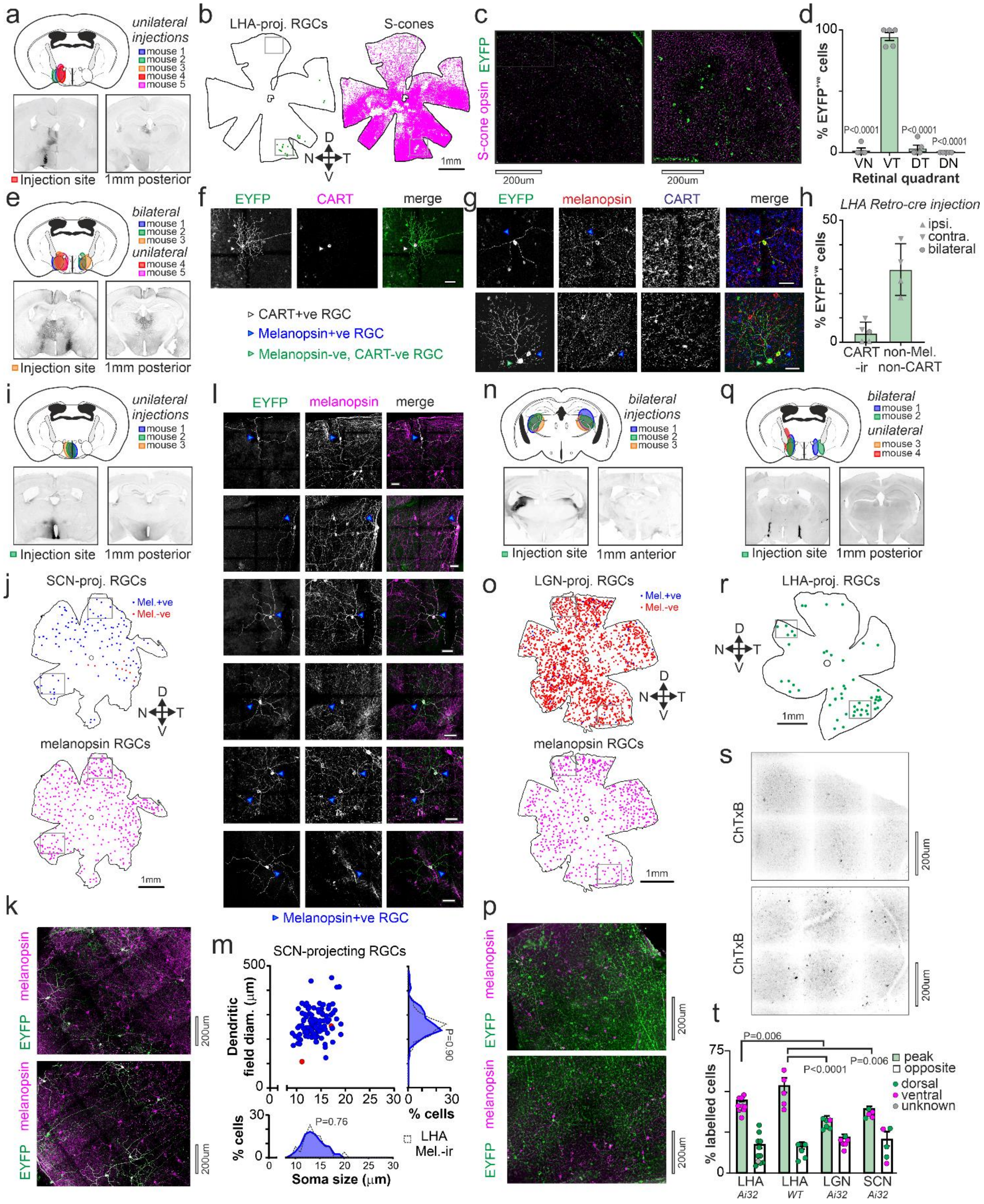

Fig S5. Characterisation of lateral hypothalamic-projecting RGCs. (**a**) Traced LHA injections sites for experiment in Fig 6a-c (top) and images of LHA injection site from mouse in Fig6a,b (left; mouse 4) and a 1mm posterior section showing no viral spillover at the level of the rostral LGN. (**b,c**) Ipsilateral retinal trace and images of retrolabelled RGCs relative to S-cone distribution (Conventions as in Fig 6 b-c). (**d**) Mean±SEM percentage of ipsilateral LHA-projecting RGCs across retinal quadrants (n=5 unilaterally-injected Ai32 mice). Data analysed by one-way RM ANOVA ($F_{1.44,5.7}$=316.9, P=0.001) with Tukey's post-tests. (**e**) LHA injections sites for experiment in Fig 6d-f

and representative images from a bilaterally-injected animal (conventions as in **a**). (**f,g**) Example CART+ve (**f**) and CART-ve/melanopsin-ve (**g**) LHA-projecting RGCs. Scale bars=50µm. (**h**) Mean±SEM percentage LHA-projecting RGCs with CART immunoreactivity (n=5 retinas) or lacking CART and melanopsin immunoreactivity (n=4/5 co-stained retinas for melanopsin). (**i, n**) Ai32$^{+/-}$ SCN (**i**) and LGN (**n**) AAVretro-Cre injections sites with representative images as in **a.** (**j,o**). Traced retinas from SCN (**j**) and LGN (**o**) injected mice showing retro-labelled and melanopsin+ RGCs. (**k**,**p**) Labelled neurons in dorsal and ventral portions of the retina, corresponding to boxed regions in **j** and **o**.(**l**) Example melanopsin+, retrolabelled SCN-projecting RGCs. Scale bars=50 µm. (**m**) Dendritic field diameter vs. soma size for SCN-projecting RGCs (n=117 cells from 6 retinas). Distributions compared against melanopsin+ LHA-projecting cells (Fig 6h) by Kolmogorov-Smirnov test. (**q**) Cholera toxin beta (ChTxB) injection sites (wildtype mice), with representative images as in **a**. (**r,s**) Traced retina (**r**) and images (**s**) showing LHA-projecting (ChtXB-labelled) RGCs (conventions as in **b**,**c**). (**t**) Labelling asymmetry across central injection sites, showing mean±SEM percentage labelled RGCs in the densest vs. opposite retinal quadrant; data points colour coded according to location in dorsal or ventral retina (where known). Data from AAVretro-Cre injections (Ai32$^{+/-}$ mice) targeting LHA (n=11 retinas), LGN (n=6 retinas) or SCN (n=5 retinas) and LHA-injections of ChtXB in WT mice (n=7 retinas) resulting in (mean+SEM): 149±36, 1326±140, 59±24 and 31±9 labelled cells/retina respectively. Data analysed by 2-way mixed effects ANOVA (QuadrantXSite: $F_{3,23}$=6.79, P=0.002; Quadrant: $F_{1,23}$=134.3, P<0.0001; Site: $F_{3,23}$= 3.247, P=0.04) with Tukey's post-tests.

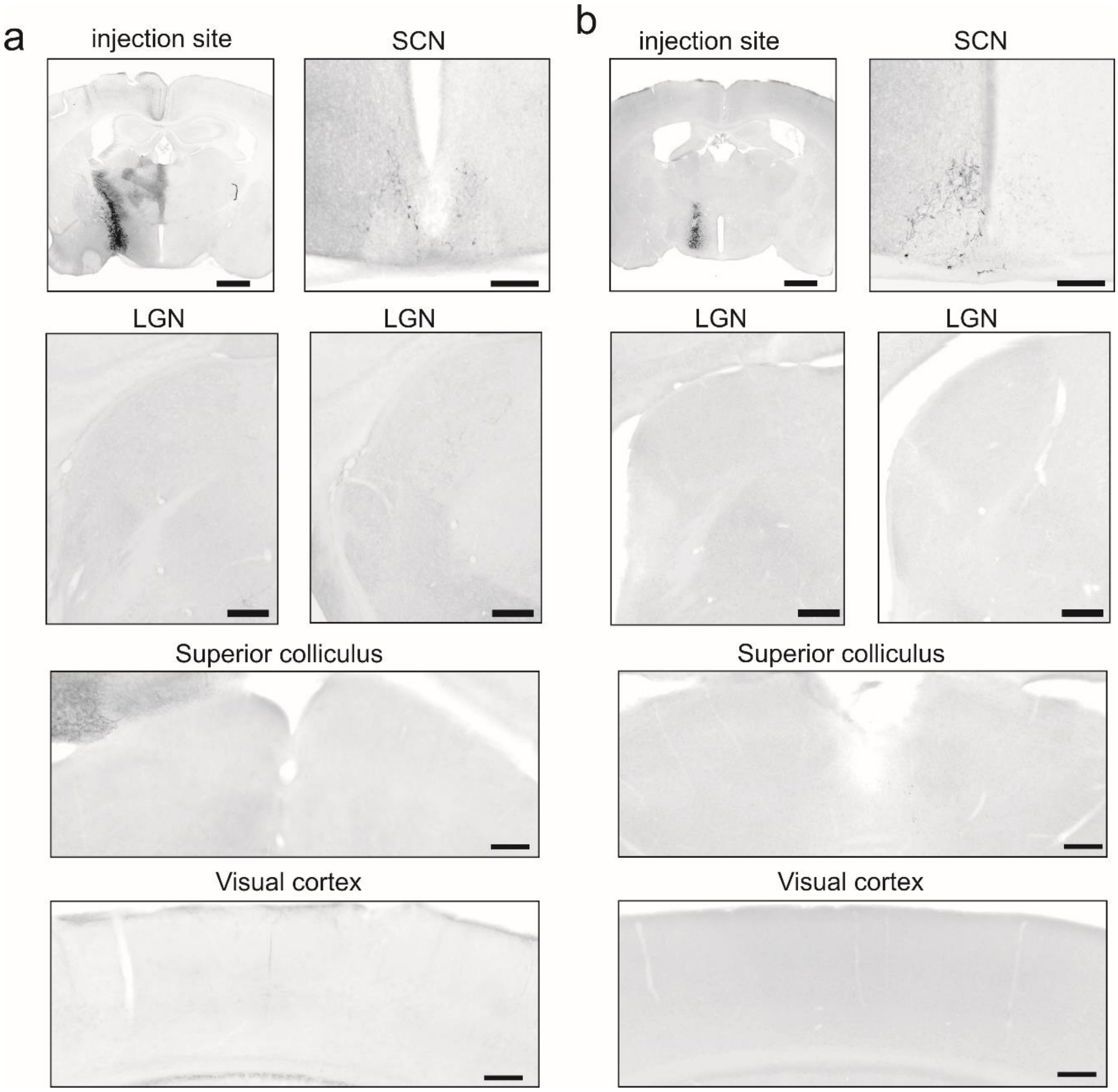

Fig S6. The LHA does not receive input from the visual thalamus or superior colliculus. (**a-b**) Representative images of EYFP labelling from two Ai32 mice receiving unilateral intra-LHA microinjections of AAVretro-hSyn-Cre. Upper panels show injection site (left) and representative images of retro-labelled neurons in the SCN (right). Mid and lower panels show absence of retro-labelling in the visual thalamus, superior colliculus and visual cortex respectively. Scale bars = 200µm except upper right panels (1mm).

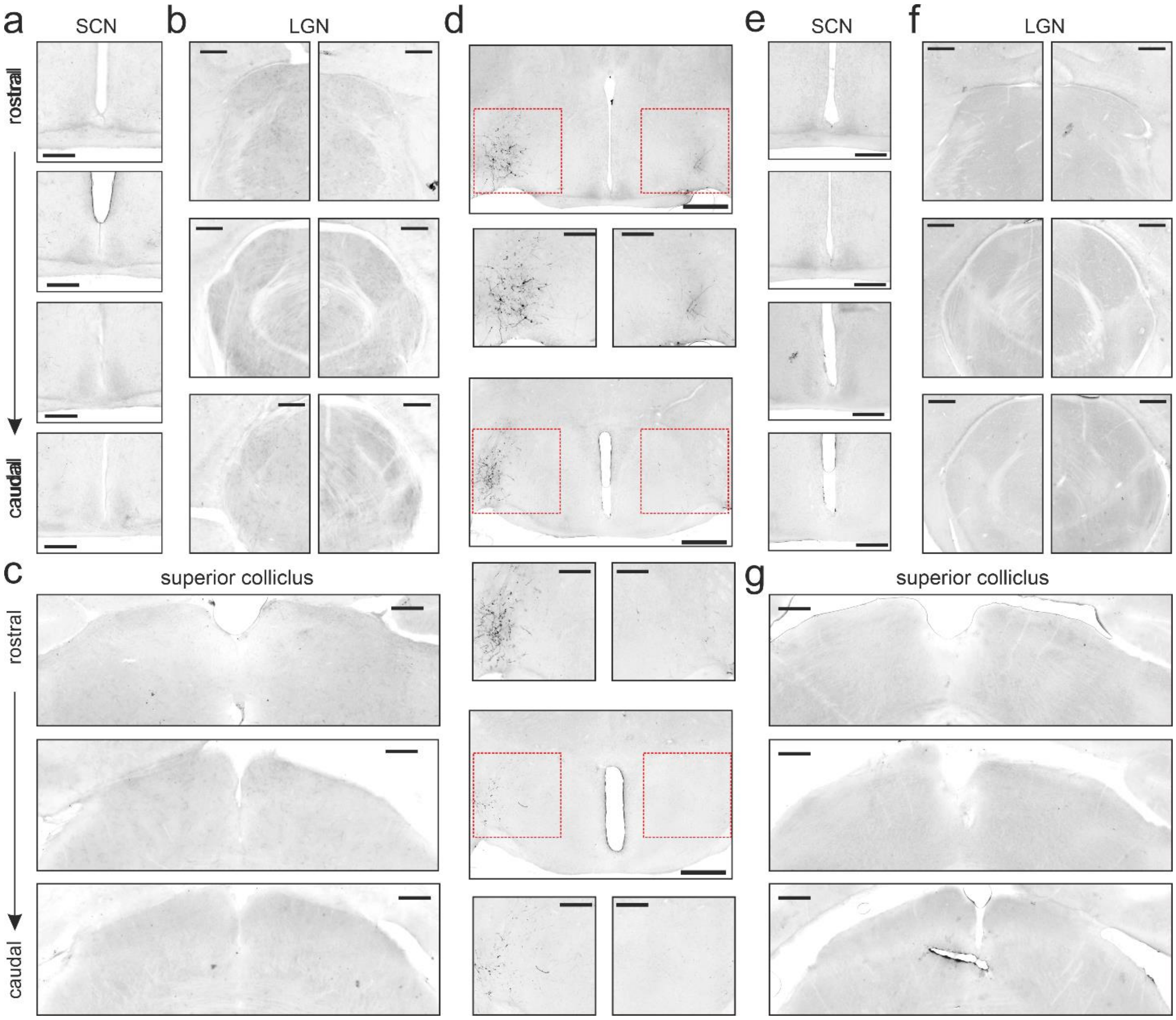

Fig S7. Negligible collateral projections of lateral hypothalamic-projecting RGCs. (**a-c**) Rostral to caudal images of the SCN (**a**), LGN (**b**) and superior colliculus (**c**) from a wildtype mouse with selective labelling of LHA-projecting RGCs (mouse shown in Fig 6j-l). (**d**) Rostral to caudal images of LHA from a second wildtype mouse with intersectional, selective, labelling of LHA-projecting RGCs as above. (**e-g**) Rostral to caudal images of the SCN (**e**), LGN (**f**) and superior colliculus (**g**) from mouse in **d**. Scale bars=500μm except **a**,**e** and insets in **d** where scale bars=250μm.

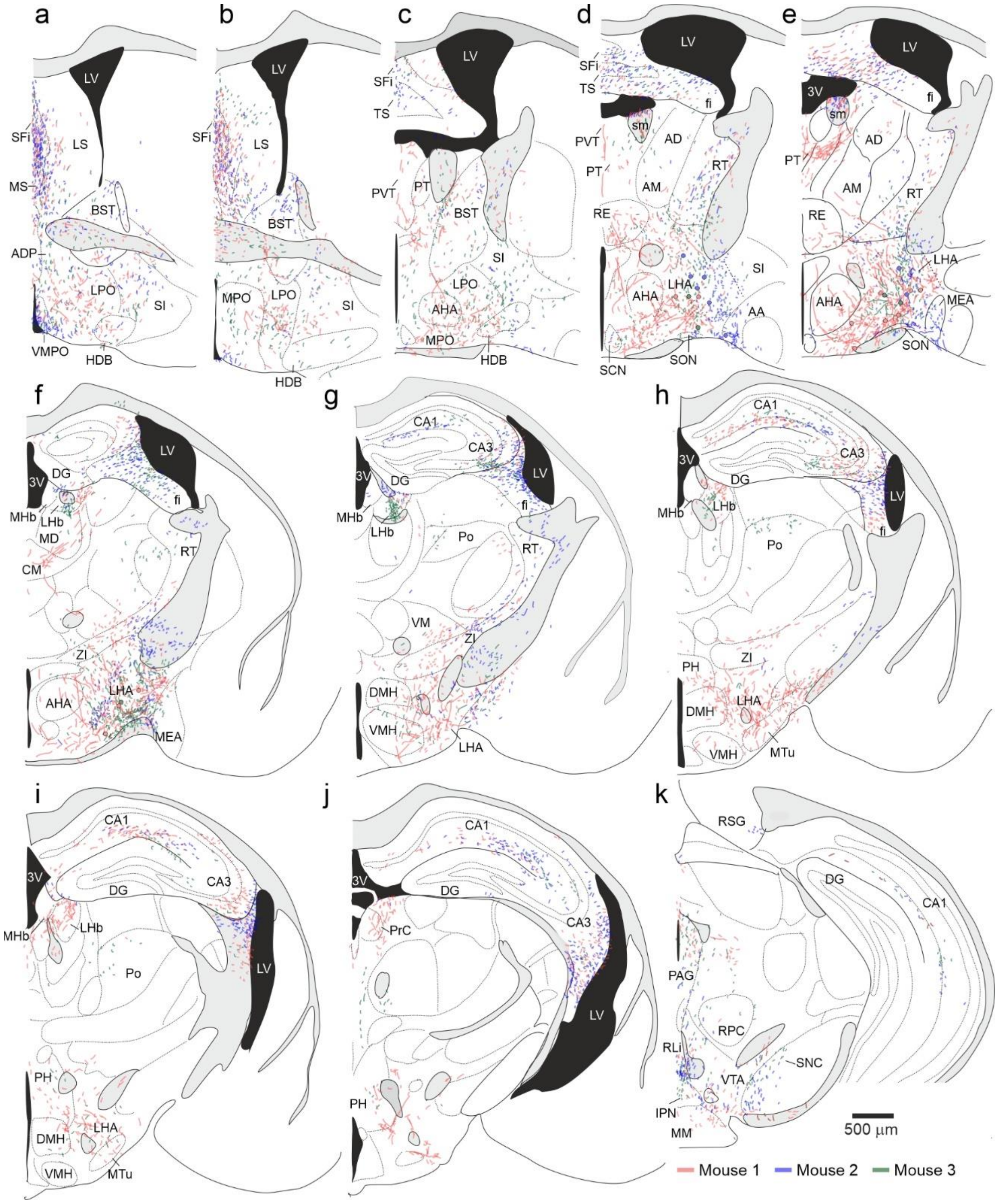

Fig S8. Projection targets of retinorecipient lateral hypothalamic neurons. (**a-k**) Rostral to caudal schematics of mouse brain showing distribution of labelled fibres from three mice that received intravitreal injection of AAV1-Cre and lateral hypothalamic microinjection of AAV2-DIO-ChR2:mCherry (left hemisphere projections traced in each case). 3V: dorsal third ventricle; AA: anterior amygdaloid area; AD: anterodorsal thalamic nucleus; ADP: anterodorsal preoptic nucleus; AHA: anterior hypothalamic area; AM: anteromedial thalamic nucleus; BST: bed nucleus of the stria terminalis; CM: centromedial thalamic nucleus; DMH: dorsomedial hypothalamic nucleus; DG: dentate gyrus; fi: fimbra of hippocampus; HDB: nucleus of the horizontal limb of the diagonal band; IPN: interpeduncular nucleus; LHb: lateral habenula; LPO: lateral preoptic area; LHA: lateral hypothalamic area; LS: lateral septal nucleus; LV: lateral ventricle; MD: mediodorsal thalamic nucleus; MEA: medial amygdaloid nucleus.; MHb: medial habenula; MM: medial mammillary nucleus; MPO: medial preoptic area; MS: medial septal nucleus; MTu: medial tuberal nucleus; PAG: periaqueductal grey; PH: posterior hypothalamus; Po: posterior thalamus; PrC: pericommissural

nucleus; PT: paratenial thalamic nucleus; PVT: paraventricular nucleus of the thalamus; RE: nucleus reuniens; RLi: rostral linear nucleus of raphe; RSG: retrosplenial granular cortex; RPC: parvicellular red nucleus; SFi: septofimbral nucleus; SI: substantia innominate; SON: supraoptic nucleus; sm: stria medullaris; SNC: substantia nigra pars compacta; ZI: zona incerta; VMPO: ventromedial preoptic nucleus; VM: ventromedial thalamic nucleus; VMH: ventromedial hypothalamic nucleus; VTA: ventral tegmental area.

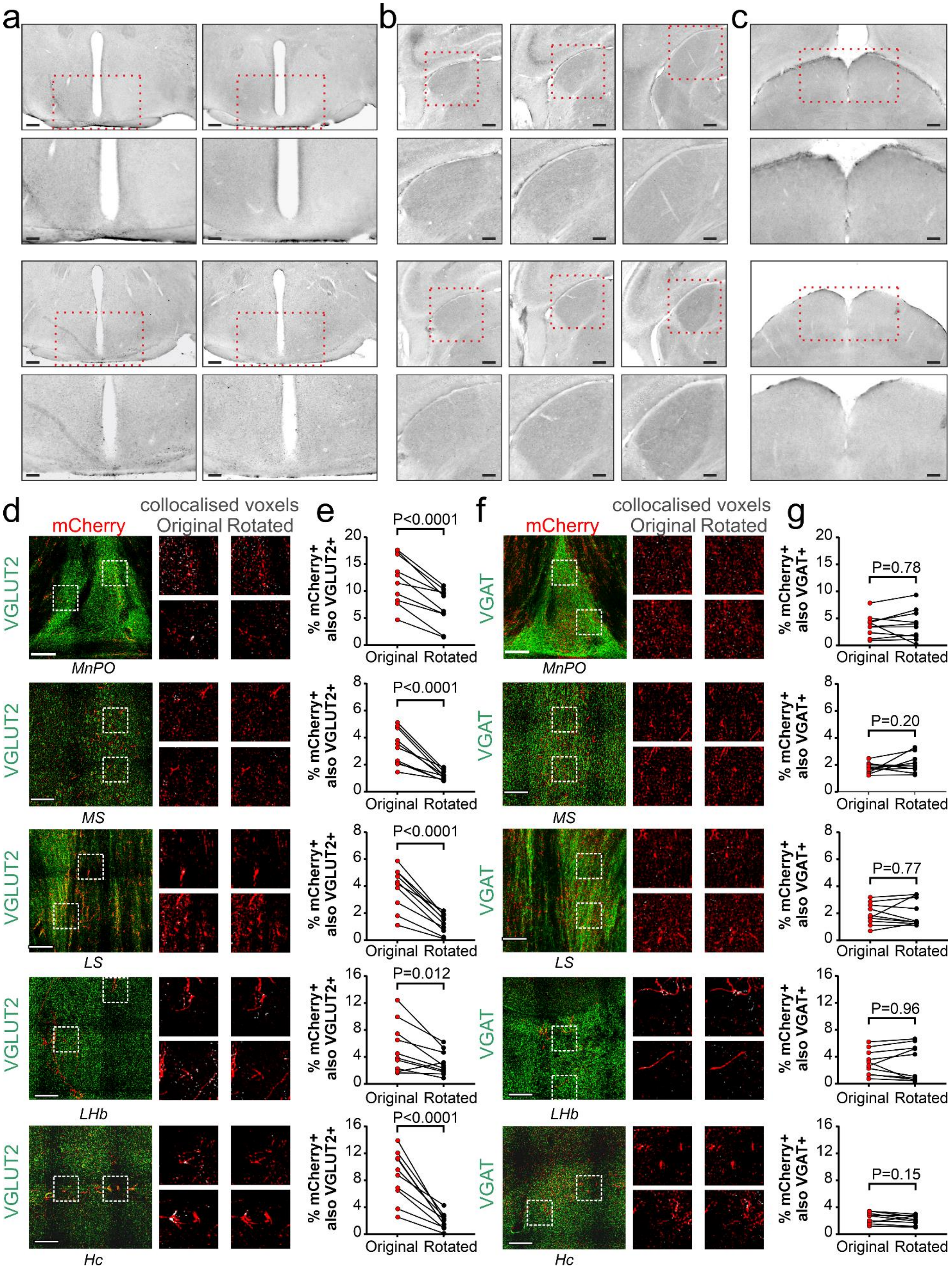

Fig S9. Co-localisation of retinorecipient lateral hypothalamic neuronal projections with glutamatergic and GABAergic markers. (**a**-**c**) Representative mCherry strained sections from two animals (upper and lower rows) with selective targeting of retinorecipient LHA neurons, showing absence of any labelled neurons in other visual targets; SCN (**a**), LGN (**b**) and superior colliculus (**c**). Scale bars are 100μm for upper macro images and 100μm for lower, zoomed, inserts. (**d,f**)

Example of ROIs analysed for co-localisation of mCherry-labelled fibres from retinorecipient LHA neurons with VLUT2 (**d**) or VGAT immunoreactivity (**f**). Left panels show confocal max-projection of mCherry+ and VGLUT+/VGAT+ voxels. Middle and right panels show mCherry+ voxels (red) and voxels co-localising with VGLUT or VGAT (white) for two example 70x70µm subfields, either for the original image (middle panels) or when the VGLUT/VGAT channel was locally rotated by 90° (right panels). Scale bars in all images=75µm. (**e,g**) Analysis of co-localisation for ROIs corresponding to the example images in **d** and **f**. In each case data points are derived from the 10 (70x70 µm) subfields from each ROI with the highest proportion of mCherry+ voxels and compare the proportion of those voxels that were also VGLUT2+ (**e**) or VGAT+ (**g**) in the original image or when the VGLUT/VGAT channel was locally rotated by 90° for each subfield. In each case, data analysed by two-tailed paired t-test.

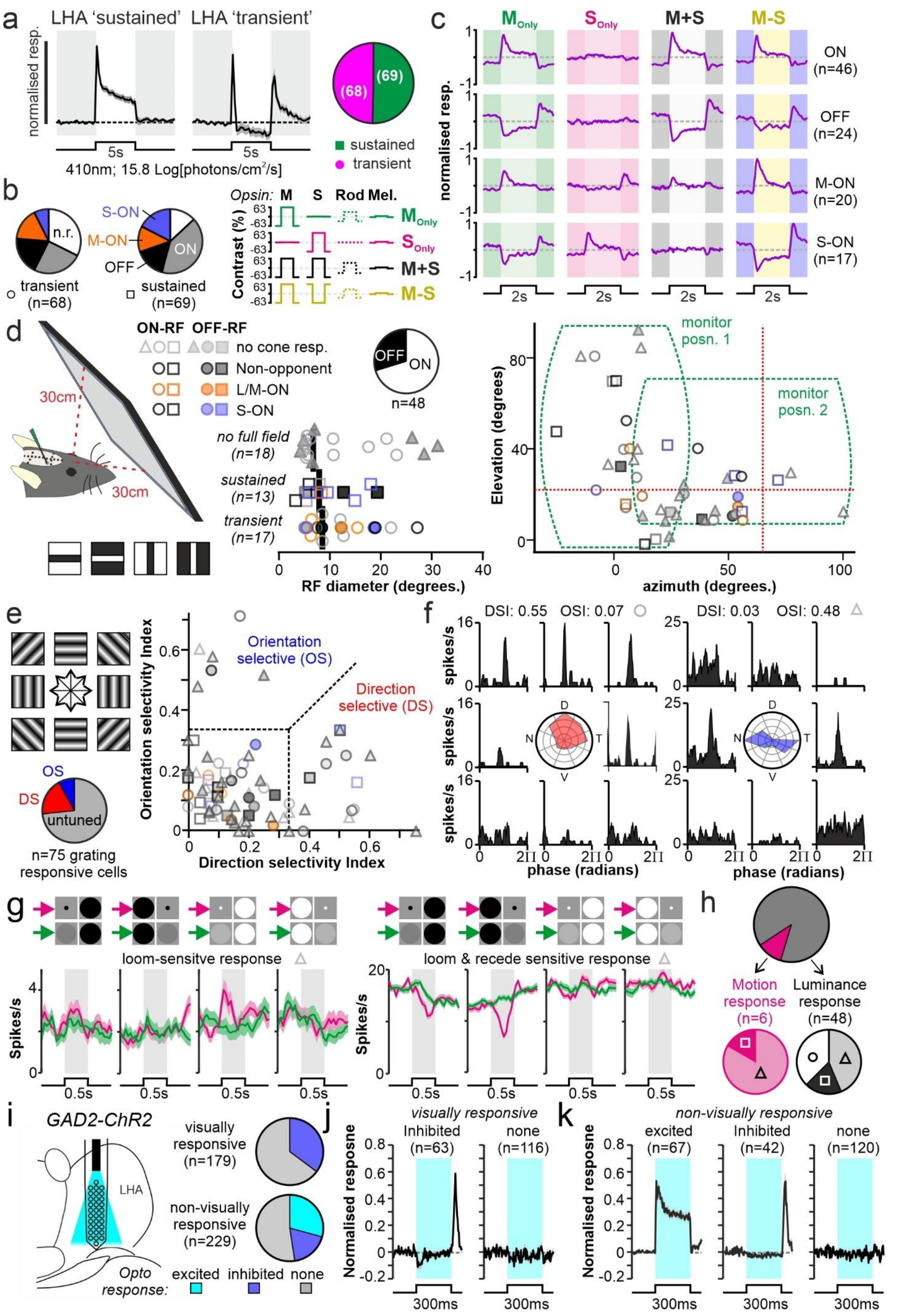

Fig S10. Visually responsive lateral hypothalamic neurons are distinct from optogenetically identifiable GABAergic neurons. (**a**) Mean±SEM normalised responses of sustained (left) and

transient (right) LHA neurons in GAD2-ChR2 mice and pie chart showing proportions detected (n=137 cells from 15 mice). (**b**) Proportions of sustained and transient LHA neurons displaying non-opponent (ON or OFF), colour opponent (M-ON or S-ON) or no response (n.r) to full-field cone-directed stimuli; stimuli presented at an irradiance of ~14.4 Log rod-effective photons/cm^2/s to mitigate rod contrasts associated with M-cone directed stimuli. (**c**) Mean±SEM normalised responses of GAD2-ChR2 LHA neurons displaying non-opponent (ON or OFF) or colour opponent (M-ON or S-ON) responses (n=46,24,20 and 17 respectively). (**d**) RF mapping in a subset GAD2-ChR2 LHA recordings (n=7 mice), showing proportions of responsive neurons with ON or OFF RFs and RF diameter as a function of response classification using full-field visual stimuli (left panels) and corresponding RF location (right); conventions as per Fig 3c. (**e**) Direction and orientation selectivity indices (DSI/OSI; stimulus set and calculations as per Fig 4a,b) for GAD2-ChR2 LHA neurons that exhibited significant responses to drifting grating stimuli (n=75 cells from 7 mice) and proportions classified as DS, OS or untuned. (**f**) Responses of representative DS (left) or OS (right) cells to gratings of optimal spatial and temporal frequency across the 8 tested direction of motion (conventions as in Fig 4d,e). (**g**) mean+SEM firing rate (from 20 trials) of two representative cells with preferential responses to approaching and/or receding spots, stimuli and conventions as per Fig 4f-k. (**h**) Proportion of responding GAD2-ChR2 LHA neurons (n=54 cells from 7 mice) with luminance or motion-sensitive responses and classification based on response to full field light steps. (**i**) Proportions of LHA cells from GAD2-ChR2 mice (n=15) with direct excitatory, inhibitory (including rebound excitation) or no response to optogenetic stimulation across visually responsive (n=179 cells) or non-visually responsive cells (n=229). (**j,k**) Mean±SEM normalised responses of visually responsive (**j**) and non-visually responsive (**k**) GAD2-ChR2 LHA neurons (300ms, 460nm flash, ~630mW/mm^2 light energy at the optrode fibre tip).

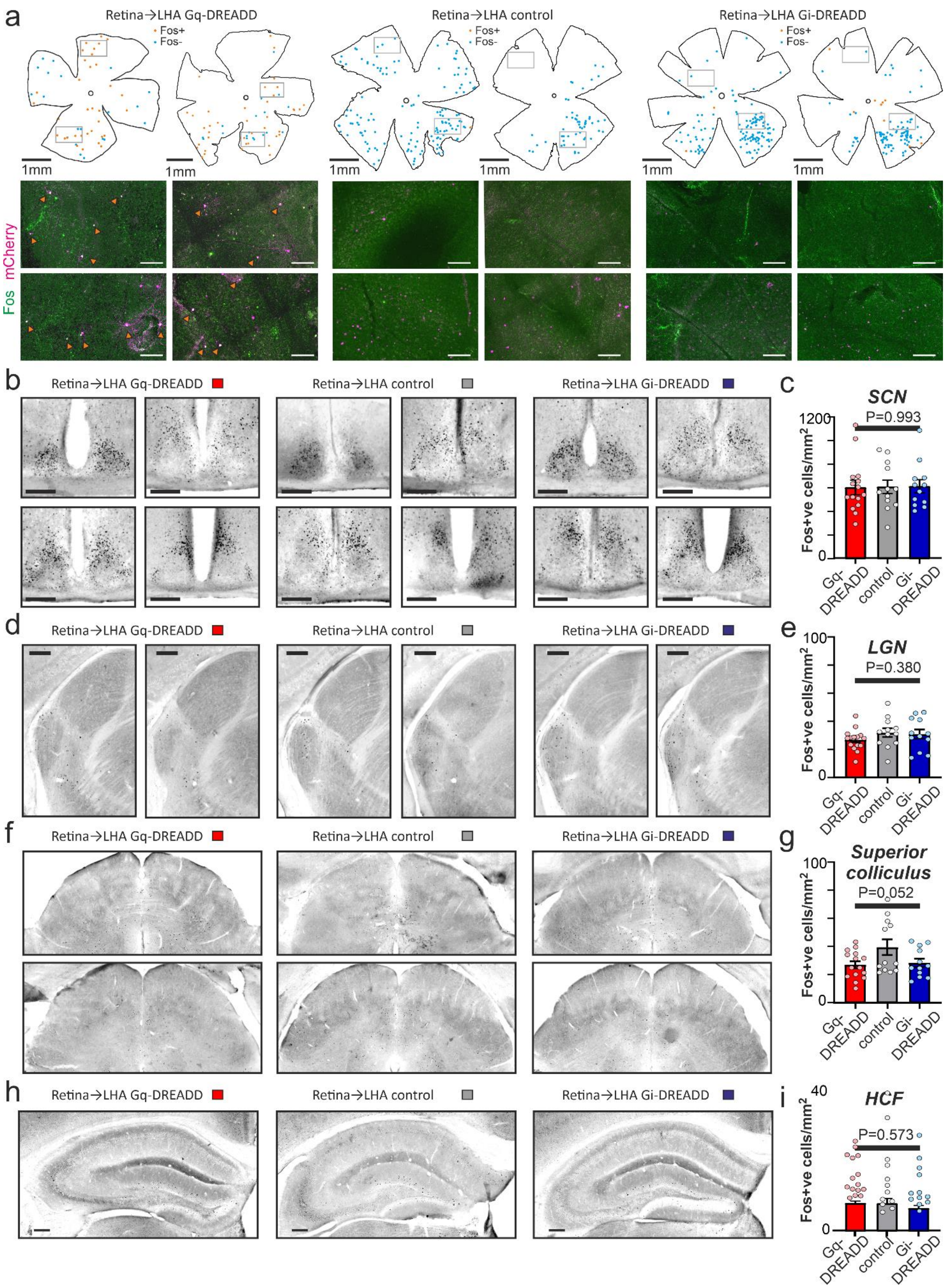

Fig S11. Chemogenetic manipulation of retinal input to the lateral hypothalamic area does not impact activity in other visual regions or the hippocampus. (**a**) Additional example whole mount retinas co-labelled for Fos and mCherry to identify LHA-projecting RGCs (left to right, Gq-DREAAD

expressing, mCherry control and Gi-DREADD expressing). Top panels show distribution of Fos+ve and Fo-ve LHA-projecting RGCs, lower panels show confocal max-projection corresponding to boxed regions in upper panels, and c-Fos, arrowheads indicate double labelled cells, scale bars = 200µm. (**b**,**d**,**f,h**) Representative images of c-Fos expression in the SCN (**b**), LGN (**d**), superior colliculus (**f**), and dorsal hippocampus (**h**) of Gq-DREAAD, control and Gi-DREADD (left- right respectively) transduced animals. Scale bars= 200 µm. (**c**,**e**,**g**,**i**) Mean±SEM density of c-Fos immunoreactive nuclei from SCN (**c**), LGN (**e**), superior colliculus (**g**), and dorsal hippocampus (**i**) across experimental groups. Data in analysed by one-way ANOVA (**c**: $F_{2, 36}$=0.007, P=0.99; e: $F_{2, 36}$=0.99, P=0.38; g: $F_{2, 36}$=3.2, P=0.052; i: $F_{2, 36}$=0.57, P=0.57).

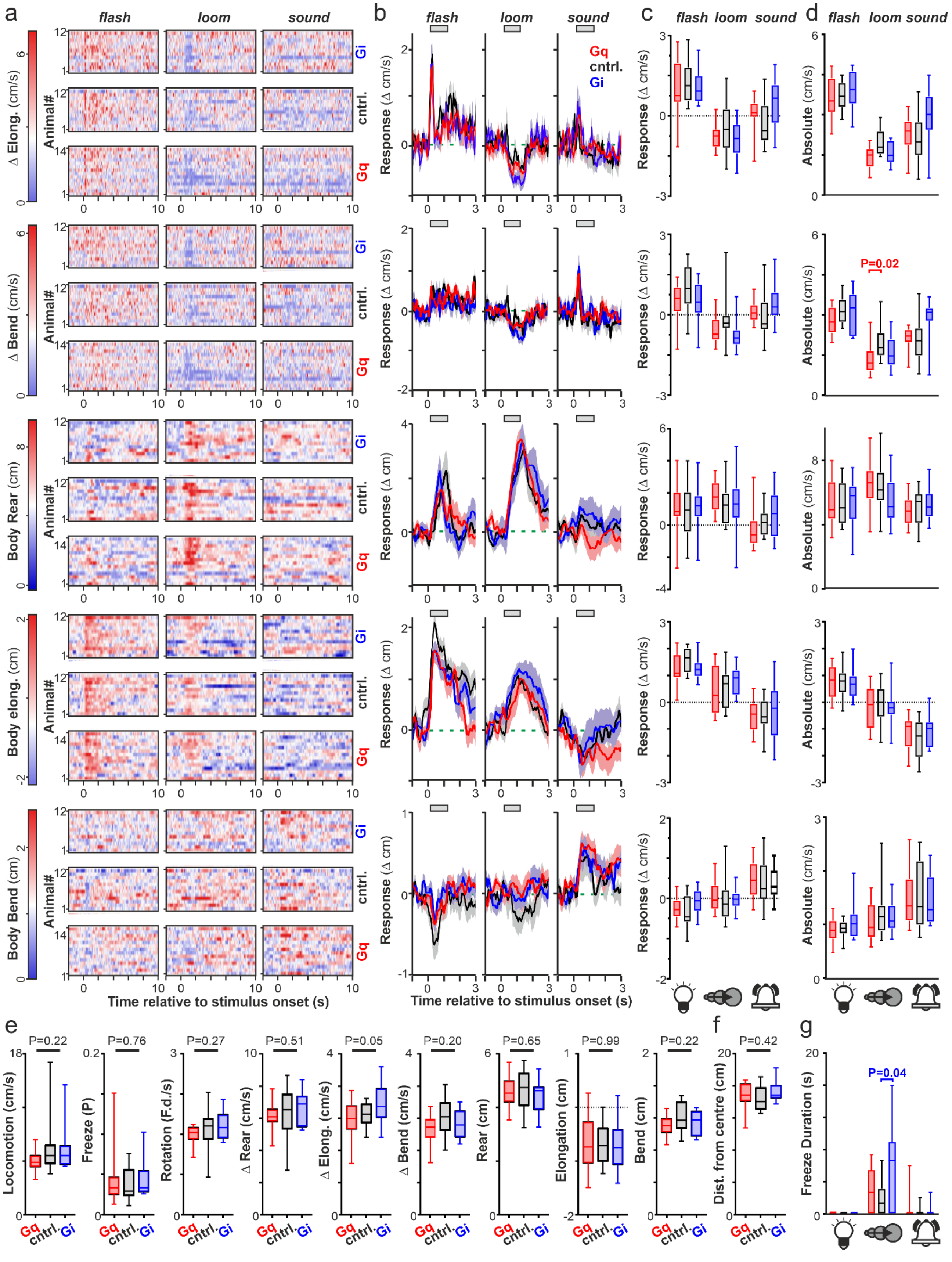

Fig S12. Retinal inputs to the lateral hypothalamic area do not overtly impact spontaneous mouse behaviour or postural responses. (**a**) Heatmaps for additional motion and postural-related behaviours across stimulus types and animal/experimental groups in Fig 9 (Gq-DREADD, n=14; Gi-DREADD, n=12; control, n=12), conventions as in Figure 9b. (**b**) Mean±SEM change in each

behavioural component (relative to 2s pre-stimulus mean) for animals and stimuli in **a.** (**c, d**) Box and whisker plots (box=quartiles 2-3,centre=median, whiskers=min-max) showing change from baseline (**c**; relative to the 2s pre-stimulus) and absolute values (**d**) for motion- and posture-related components from **a**-**b**. (**e, f**) Box and whisker plots, as above, for the 6 movement and 3 postural behavioural measures (as illustrated in Fig 9a) and distance from centre of behavioural area (**b**) in the same Retina→LHA Gq-DREADD (n=14), Gi-DREADD (n=12) and control (n=12) mice during spontaneous activity in dim light. (**g**) stimulus-evoked freezing bout duration (see Methods) for the mice above following flash, loom and auditory stimuli. Data in **c**, **d** and **g** analysed by 2-way mixed effects ANOVA, with stimulus type as within-subjects variable (**c**: *Δ Elongation:* DREADDXStimulus-$F_{4,70}$=1.9, P=0.12; DREADD-$F_{2,35}$=0.31, P=0.73, Stimulus- $F_{1.8,62}$=47.8, P<0.0001; *Δ Bend:* DREADDXStimulus-$F_{4,70}$=1.9, P=0.12; DREADD-$F_{2,35}$=0.99, P=0.37, Stimulus-$F_{1.9,65}$=24.3, P<0.0001; *Rear:* DREADDXStimulus-$F_{4,70}$=0.57, P=0.68; DREADD- $F_{2,35}$=0.36, P=0.70, Stimulus-$F_{1.8,63}$=5.7, P=0.007; *Elongation:* DREADDXStimulus-$F_{4,70}$=0.63, P=0.64; DREADD-$F_{2,35}$=0.70, P=0.50, Stimulus-$F_{2.0,69}$=68.6, P<0.0001; *Bend:* DREADDXStimulus- $F_{4,70}$=0.30, P=0.88; DREADD-$F_{2,35}$=1.06, P=0.36, Stimulus-$F_{2.0,69}$=23.6, P<0.0001; **d**: *Δ Elongation:* DREADDXStimulus-$F_{4,70}$=2.48, P=0.052; DREADD-$F_{2,35}$=2.90, P=0.07, Stimulus-$F_{1.7,59}$=91.0, P<0.0001; *Δ Bend:* DREADDXStimulus-$F_{4,70}$=2.93, P=0.03; DREADD-$F_{2,35}$=4.11, P=0.02, Stimulus- $F_{1.9,65}$=50.8, P<0.0001; *Rear:* DREADDXStimulus-$F_{4,70}$=1.16, P=0.33; DREADD-$F_{2,35}$=0.53, P=0.60, Stimulus-$F_{1.8,65}$=6.8, P=0.003; *Elongation* DREADDXStimulus-$F_{4,70}$=0.58, P=0.68; DREADD- $F_{2,35}$=0.03, P=0.97, Stimulus-$F_{1.8,64}$=107.9, P<0.0001; *Bend:* DREADDXStimulus-$F_{4,70}$=0.44, P=0.77; DREADD $F_{2,35}$=0.33, P=0.71, Stimulus-$F_{1.6,57}$=16.8, P<0.0001; **g:** *Freeze duration*: DREADDXStimulus-$F_{4,70}$=3.98, P=0.006; DREADD-$F_{2,35}$=3.13, P=0.07, Stimulus-$F_{1.2,43}$=30.8, P<0.0001). Dunnett's post-tests between Gq-DREADD/control and Gi-DREADD/control were applied for each stimulus type, wherever ANOVA revealed a significant effect of DREADDXStimulus; statistically significant differences indicated on the relevant plots, otherwise P>0.05. Data in **e** and **f** analysed by one-way ANOVA (**e**: *Locomotion*: $F_{2,35}$=1.59, P=0.22; *Freeze probability*: $F_{2,35}$=0.28, P=0.76; *Rotation:* $F_{2,35}$=1.37, P=0.27; *Δ Rear*: $F_{2,35}$=0.70, P=0.51; *Δ Elongation*: $F_{2,35}$=3.20, P=0.052; *Δ Bend*: $F_{2,35}$=1.70, P=0.20; *Rear:* $F_{2,35}$=0.44, P=0.65; *Elongation:* $F_{2,35}$=0.01, P=0.99; *Bend:* $F_{2,35}$=1.59, P=0.22; **f:** *Distance from centre*: $F_{2,35}$=0.90, P=0.42).

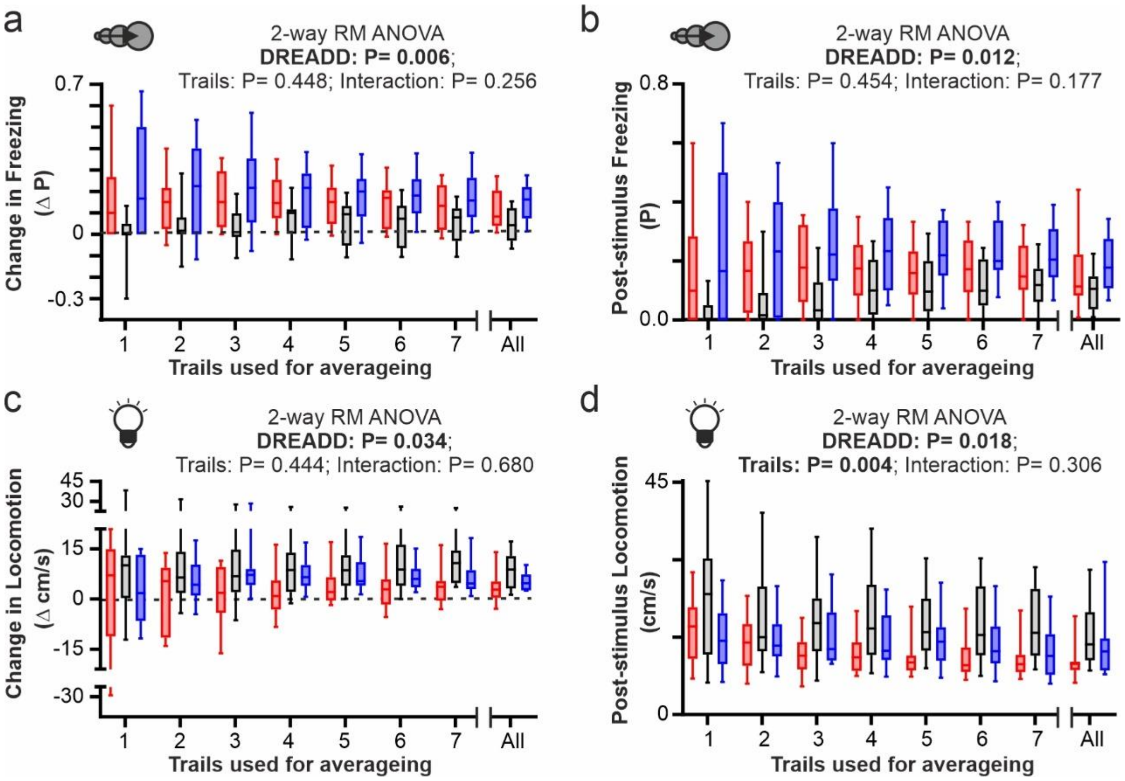

Fig S13. Influence of trail-averaging changes in mouse visually guided behaviour following chemogenetic manipulation of RGC inputs to the LHA. (**a-d**) Bow and whisker plots (quartiles 2-3 and min-max respectively) showing change from baseline (**a, c**; relative to the 2s preceding stimulus) and absolute values (**b, d**) for looming induced freezing (**a-b**) and flash-induced locomotion (**c-d**) across Gq DREADD, Gi-DREAD and control mice (n=14,12 and 12 respectively) as a function of number of trials included in averaging, starting from eth first exposure to each stimulus type. Data analysed by 2-way mixed effects ANOVA with trails as a within subjects variable (**a**: DREADD- $F_{2, 35}$ = 5.88; P=0.006; Trials- $F_{2.6, 91.7}$ = 0.87; P=0.448; DREADDXTrials$_{14, 245}$ = 1.2; P=0.256; **b**: DREADD- $F_{2, 35}$ = 5.05; P=0.012; Trials- $F_{2.4, 82.4}$ = 0.84; P=0.454; DREADDXTrials$_{14, 245}$ = 1.4; P=0.177; **c**: DREADD- $F_{2, 35}$ = 3.73; P=0.034; Trials- $F_{1.9, 66.5}$ = 0.81; P=0.444; DREADDXTrials$_{14, 245}$ = 0.8; P=0.68; **d**: DREADD- $F_{2, 35}$ = 4.53; P=0.018; Trials- $F_{1.9, 65.8}$ = 9.19; P=0.004; DREADDXTrials$_{14, 245}$ = 1.2; P=0.306).

General information					Data from light responsive (LR) channels							Data from non-LR channels	
					Full field stimuli					All Stimuli			
Type	Subset	Data Fig.	No. Mice	No. Plcmnt.	Chans.	Total units	Sus.	Tran.	N.R.	Resp	N.R.	Chans.	Units
Opn1mwR LHA	All	Fig 1, Fig S1	20	68	179	*N.A.*						1997	
	spike sorted data	Fig 1, Figs S1-S2	18	18	146	62	19	25	18	*N.A.*		430	86
Opn1mwR SCN	All	Fig 1, Figs S1-S2	15	15	81	57	39	10	8	*N.A.*		399	203
C57 LHA	All	Fig 1, Fig S1	18	18	136	51	19	15	17	*N.A.*		440	36
Opn1mwR LHA	All	Figs 2-3, Figs S2-3	9	9	252	237	61	82	94	*N.A.*		36	8
	with spatial stimuli	Figs 3-5, FigS3-4	5	5	160	188	50	66	72	178	10	0	0
GAD2-ChR2 LHA	All	Fig S10	15	15	270	274	69	68	137	*N.A.*		210	134
	with spatial stimuli	Fig S10	7	7	145	132	30	32	70	104	28	79	63

Supplemental Table 1. Details of Electrophysiological Recordings. Details of the number of mice, recordings and responsive or non-responsive (NR) units across Light responsive and non-light responsive channels.
