# Supplementary material for: A lateral hypothalamic region supporting diverse visual processing and modulation of visually-guided behaviour
Source: Nat Commun. 2025 Nov 11;16:9917. doi: 10.1038/s41467-025-64864-3 (PMC12606360; doi:10.1038/s41467-025-64864-3)
Supplement: Supplementary file 2 — Reporting Summary [file 41467_2025_64864_MOESM2_ESM.pdf]

nature portfolio

Corresponding author(s): Timothy Brown
Last updated by author(s): 12/09/2025

Reporting Summary

Nature Portfolio wishes to improve the reproducibility of the work that we publish. This form provides structure for consistency and transparency in reporting. For further information on Nature Portfolio policies, see our Editorial Policies and the Editorial Policy Checklist.

Statistics

For all statistical analyses, confirm that the following items are present in the figure legend, table legend, main text, or Methods section.

n/a	Confirmed	
☐	☒	The exact sample size (*n*) for each experimental group/condition, given as a discrete number and unit of measurement
☐	☒	A statement on whether measurements were taken from distinct samples or whether the same sample was measured repeatedly
☐	☒	The statistical test(s) used AND whether they are one- or two-sided<br>*Only common tests should be described solely by name; describe more complex techniques in the Methods section.*
☒	☐	A description of all covariates tested
☐	☒	A description of any assumptions or corrections, such as tests of normality and adjustment for multiple comparisons
☐	☒	A full description of the statistical parameters including central tendency (e.g. means) or other basic estimates (e.g. regression coefficient) AND variation (e.g. standard deviation) or associated estimates of uncertainty (e.g. confidence intervals)
☐	☒	For null hypothesis testing, the test statistic (e.g. *F*, *t*, *r*) with confidence intervals, effect sizes, degrees of freedom and *P* value noted<br>*Give P values as exact values whenever suitable.*
☒	☐	For Bayesian analysis, information on the choice of priors and Markov chain Monte Carlo settings
☒	☐	For hierarchical and complex designs, identification of the appropriate level for tests and full reporting of outcomes
☒	☐	Estimates of effect sizes (e.g. Cohen's *d*, Pearson's *r*), indicating how they were calculated

Our web collection on statistics for biologists contains articles on many of the points above.

Software and code

Policy information about availability of computer code

Data collection	Recorder64 (Plexon, TX, USA)<br>LabVIEW 2019 (National Instruments, TX, USA)<br>Psychophysics Toolbox v3 (http://psychtoolbox.org/)
Data analysis	Offline Sorter V.3.3.5 (Plexon, TX, USA)<br>Matlab R2017a (Mathworks, MD, USA)<br>Kilosort V2 (dx.doi.org/10.1101/061481)<br>ImageJ v1.52a (NIH, MD, USA)<br>CaseViewer v2.4 (3DHISTECH, Budapest, Hungary)<br>HighDimDefneseBehaviours Toolbox (https://doi.org/10.5281/zenodo.13331479; https://github.com/RStorchi/HighDimDefenseBehaviours)<br>Graphpad Prism v9.3.1 (GraphPad Software, CA, USA)

For manuscripts utilizing custom algorithms or software that are central to the research but not yet described in published literature, software must be made available to editors and reviewers. We strongly encourage code deposition in a community repository (e.g. GitHub). See the Nature Portfolio guidelines for submitting code & software for further information.

Data

Policy information about availability of data

All manuscripts must include a data availability statement. This statement should provide the following information, where applicable:

- Accession codes, unique identifiers, or web links for publicly available datasets
- A description of any restrictions on data availability
- For clinical datasets or third party data, please ensure that the statement adheres to our policy

Data underlying all quantitative analysis that supports the findings of this study are provided in the Source data accompanying this paper. Other raw data is available from the corresponding author on request. Source data are provided with this paper.

Research involving human participants, their data, or biological material

Policy information about studies with human participants or human data. See also policy information about sex, gender (identity/presentation), and sexual orientation and race, ethnicity and racism.

Reporting on sex and gender	NA
Reporting on race, ethnicity, or other socially relevant groupings	NA
Population characteristics	NA
Recruitment	NA
Ethics oversight	NA

Note that full information on the approval of the study protocol must also be provided in the manuscript.

Field-specific reporting

Please select the one below that is the best fit for your research. If you are not sure, read the appropriate sections before making your selection.

☒ Life sciences ☐ Behavioural & social sciences ☐ Ecological, evolutionary & environmental sciences

For a reference copy of the document with all sections, see nature.com/documents/nr-reporting-summary-flat.pdf

Life sciences study design

All studies must disclose on these points even when the disclosure is negative.

Sample size	Experimental design throughout was informed by previous studies performed in the investigators laboratories and corresponding power analyses performed using G*power 3.1 (Universitat Kiel, Germany). Sample sizes were set such that effect sizes (d) of 2-2.5 could be identified at power=0.95 and alpha=0.05.
Data exclusions	Data exclusions were limited to C-Fos and behavioral studies (Figs 8 and 9 in the manuscript) where 8/48 mice were excluded due to failed injections and 1 mouse was excluded due to an error in stimulus deliver. Exclusions were performed blind to experimental group and are described in full in the manuscript.
Replication	All experiments involved multiple experimental animals and key electrophysiological, and neuroanatomical findings were replicated in multiple (2 or more) independent experiments. All attempts at replication were successful.
Randomization	Throughout experimental stimuli were presented in pseudorandom order. Where relevant (C-Fos and behavioral studies; Figs 8 and 9 in the manuscript), animals were randomly assigned to experimental groups.
Blinding	Wherever relevant (cFos expression in Figure 8), analysis was performed blind to experimental group. Other analyses used objective, automated, proceedures (Fig 9) as described in the manuscript or did not involve between group comparisons.

Reporting for specific materials, systems and methods

We require information from authors about some types of materials, experimental systems and methods used in many studies. Here, indicate whether each material, system or method listed is relevant to your study. If you are not sure if a list item applies to your research, read the appropriate section before selecting a response.

Materials & experimental systems

n/a	Involved in the study
☐	☒ Antibodies
☐	☐ Eukaryotic cell lines
☐	☐ Palaeontology and archaeology
☐	☒ Animals and other organisms
☐	☐ Clinical data
☐	☐ Dual use research of concern
☐	☐ Plants

Methods

n/a	Involved in the study
☐	☐ ChIP-seq
☐	☐ Flow cytometry
☐	☐ MRI-based neuroimaging

Antibodies

Antibodies used

Chicken anti-GFP (A10262, 1:1000; Abcam, MA, USA
Rabbit anti-S opsin (AB5407, 1:500; Sigma Aldritch)
Rabbit anti-melanopsin (AB-N39, 1:500, Stratech, Ely, UK)
Rabbit anti-CART(H-003-62, 1:1000; Pheonic Pharmceuticals Inc, CA, USA)
Goat anti-CART (AF163, 1:1000; Bio-techne, MN, USA)
Chicken anti-mCherry (ab205402, 1:1000; Abcam)
Rabbit anti-VGAT (131-002, 1:1000; Synaptic Systems, Goettingen)
Rabbit anti-VGLUT2 (AGC-036, 1:1000; Alomone Labs, Jerusalem, Israel)
Rabbit anti-cFos (ab190289, 1:1000; Abcam)
Alexa 488 goat anti-chicken (A32931, 1:500; Thermo Fisher Scientific, MA, USA).
Alexa-488 donkey anti-rabbit (A21206, 1:500; Thermo Fisher Scientific).
Alexa-555 goat anti-chicken (ab150174, 1:500; Abcam).
Alexa-555 donkey anti-rabbit (A21428, 1:500; Thermo Fisher Scientific).
Alexa-647 donkey anti-goat (A21447, 1:500; Thermo Fisher Scientific).

Validation

All antibodies have been extensively used/validated previously as outlined on the manufacturers websites::
https://www.abcam.com
https://www.thermofisher.com
https://www.stratech.co.uk
https://www.sigmaaldritch.com
https://www.bio-techne.com
https://www.sysy.com
https://www.alomone.com
https://www.pheonixpeptide.com

Eukaryotic cell lines

Policy information about cell lines and Sex and Gender in Research

Cell line source(s)	NA
Authentication	NA
Mycoplasma contamination	NA
Commonly misidentified lines (See ICLAC register)	NA

Palaeontology and Archaeology

Specimen provenance	NA
Specimen deposition	NA
Dating methods	NA

☐ Tick this box to confirm that the raw and calibrated dates are available in the paper or in Supplementary Information.

Ethics oversight	NA

Note that full information on the approval of the study protocol must also be provided in the manuscript.

Animals and other research organisms

Policy information about studies involving animals; ARRIVE guidelines recommended for reporting animal research, and Sex and Gender in Research

Laboratory animals	Human L-cone opsin knockin (Opn1mwR; doi:10.1073/pnas.1934712100) – male 2-5 month old<br>Human L-cone opsin knockin mice; melanopsin knockout mice (Opn1mwR; Opn4-/-; doi:10.1016/j.cub.2012.04.039) – male 2-5 month old<br>Cre-dependent ChR2-EYFP reporter mice (Ai32; JAX #012569) male and female 4-5month old<br>GAD2-driven ChR2-EYFP mice (GAD2-IRES-Cre; JAX #010802 crossed with Ai32; JAX #012569) - male 2-5 month old<br>Wildtype mice (C57BL/6NCrl; Envigo, UK) – female 2-3 month old
Wild animals	NA
Reporting on sex	Electrophysiological recordings were performed on male mice since generating male L-cone knockin experimental animals allows more efficient colony management and overall reduced animal use (given X-linking of the M/L-cone opsins). Neuoanatomical and chemogenetic studies used female mice since females can be group housed following viral injection, improving welfare (in our experience male mice must be singly housed to avoid fighting and associated impaired recovery from surgical proceedures).
Field-collected samples	NA
Ethics oversight	All animals were used in accordance with the Animals, Scientific Procedures, Act of 1986 (UK) and received both University of Manchester ethics committee and UK Home Office approval.

Note that full information on the approval of the study protocol must also be provided in the manuscript.

Clinical data

Policy information about clinical studies

All manuscripts should comply with the ICMJE guidelines for publication of clinical research and a completed CONSORT checklist must be included with all submissions.

Clinical trial registration	NA
Study protocol	NA
Data collection	NA
Outcomes	NA

Dual use research of concern

Policy information about dual use research of concern

Hazards

Could the accidental, deliberate or reckless misuse of agents or technologies generated in the work, or the application of information presented in the manuscript, pose a threat to:

No	Yes	
☒	☐	Public health
☒	☐	National security
☒	☐	Crops and/or livestock
☒	☐	Ecosystems
☒	☐	Any other significant area

Experiments of concern

Does the work involve any of these experiments of concern:

No	Yes	
☐	☐	Demonstrate how to render a vaccine ineffective
☐	☐	Confer resistance to therapeutically useful antibiotics or antiviral agents
☐	☐	Enhance the virulence of a pathogen or render a nonpathogen virulent
☐	☐	Increase transmissibility of a pathogen
☐	☐	Alter the host range of a pathogen
☐	☐	Enable evasion of diagnostic/detection modalities
☐	☐	Enable the weaponization of a biological agent or toxin
☐	☐	Any other potentially harmful combination of experiments and agents

Plants

Seed stocks

Report on the source of all seed stocks or other plant material used. If applicable, state the seed stock centre and catalogue number. If plant specimens were collected from the field, describe the collection location, date and sampling procedures.

Novel plant genotypes

Describe the methods by which all novel plant genotypes were produced. This includes those generated by transgenic approaches, gene editing, chemical/radiation-based mutagenesis and hybridization. For transgenic lines, describe the transformation method, the number of independent lines analyzed and the generation upon which experiments were performed. For gene-edited lines, describe the editor used, the endogenous sequence targeted for editing, the targeting guide RNA sequence (if applicable) and how the editor was applied.

Authentication

Describe any authentication procedures for each seed stock used or novel genotype generated. Describe any experiments used to assess the effect of a mutation and, where applicable, how potential secondary effects (e.g. second site T-DNA insertions, mosiacism, off-target gene editing) were examined.

ChIP-seq

Data deposition

☐ Confirm that both raw and final processed data have been deposited in a public database such as GEO.

☐ Confirm that you have deposited or provided access to graph files (e.g. BED files) for the called peaks.

Data access links
May remain private before publication.

For "Initial submission" or "Revised version" documents, provide reviewer access links. For your "Final submission" document, provide a link to the deposited data.

Files in database submission

Provide a list of all files available in the database submission.

Genome browser session
(e.g. UCSC)

Provide a link to an anonymized genome browser session for "Initial submission" and "Revised version" documents only, to enable peer review. Write "no longer applicable" for "Final submission" documents.

Methodology

Replicates

Describe the experimental replicates, specifying number, type and replicate agreement.

Sequencing depth

Describe the sequencing depth for each experiment, providing the total number of reads, uniquely mapped reads, length of reads and whether they were paired- or single-end.

Antibodies

Describe the antibodies used for the ChIP-seq experiments; as applicable, provide supplier name, catalog number, clone name, and lot number.

Peak calling parameters

Specify the command line program and parameters used for read mapping and peak calling, including the ChIP, control and index files used.

Data quality

Describe the methods used to ensure data quality in full detail, including how many peaks are at FDR 5% and above 5-fold enrichment.

Software

Describe the software used to collect and analyze the ChIP-seq data. For custom code that has been deposited into a community repository, provide accession details.

Flow Cytometry

Plots

Confirm that:

☐ The axis labels state the marker and fluorochrome used (e.g. CD4-FITC).

☐ The axis scales are clearly visible. Include numbers along axes only for bottom left plot of group (a 'group' is an analysis of identical markers).

☐ All plots are contour plots with outliers or pseudocolor plots.

☐ A numerical value for number of cells or percentage (with statistics) is provided.

Methodology

Sample preparation	*Describe the sample preparation, detailing the biological source of the cells and any tissue processing steps used.*
Instrument	*Identify the instrument used for data collection, specifying make and model number.*
Software	*Describe the software used to collect and analyze the flow cytometry data. For custom code that has been deposited into a community repository, provide accession details.*
Cell population abundance	*Describe the abundance of the relevant cell populations within post-sort fractions, providing details on the purity of the samples and how it was determined.*
Gating strategy	*Describe the gating strategy used for all relevant experiments, specifying the preliminary FSC/SSC gates of the starting cell population, indicating where boundaries between "positive" and "negative" staining cell populations are defined.*

☐ Tick this box to confirm that a figure exemplifying the gating strategy is provided in the Supplementary Information.

Magnetic resonance imaging

Experimental design

Design type	*Indicate task or resting state; event-related or block design.*
Design specifications	*Specify the number of blocks, trials or experimental units per session and/or subject, and specify the length of each trial or block (if trials are blocked) and interval between trials.*
Behavioral performance measures	*State number and/or type of variables recorded (e.g. correct button press, response time) and what statistics were used to establish that the subjects were performing the task as expected (e.g. mean, range, and/or standard deviation across subjects).*

Acquisition

Imaging type(s)	*Specify: functional, structural, diffusion, perfusion.*
Field strength	*Specify in Tesla*
Sequence & imaging parameters	*Specify the pulse sequence type (gradient echo, spin echo, etc.), imaging type (EPI, spiral, etc.), field of view, matrix size, slice thickness, orientation and TE/TR/flip angle.*
Area of acquisition	*State whether a whole brain scan was used OR define the area of acquisition, describing how the region was determined.*

Diffusion MRI ☐ Used ☐ Not used

Preprocessing

Preprocessing software	*Provide detail on software version and revision number and on specific parameters (model/functions, brain extraction, segmentation, smoothing kernel size, etc.).*
Normalization	*If data were normalized/standardized, describe the approach(es): specify linear or non-linear and define image types used for transformation OR indicate that data were not normalized and explain rationale for lack of normalization.*
Normalization template	*Describe the template used for normalization/transformation, specifying subject space or group standardized space (e.g. original Talairach, MNI305, ICBM152) OR indicate that the data were not normalized.*
Noise and artifact removal	*Describe your procedure(s) for artifact and structured noise removal, specifying motion parameters, tissue signals and physiological signals (heart rate, respiration).*
Volume censoring	*Define your software and/or method and criteria for volume censoring, and state the extent of such censoring.*

Statistical modeling & inference

Model type and settings | *Specify type (mass univariate, multivariate, RSA, predictive, etc.) and describe essential details of the model at the first and second levels (e.g. fixed, random or mixed effects; drift or auto-correlation).*

Effect(s) tested | *Define precise effect in terms of the task or stimulus conditions instead of psychological concepts and indicate whether ANOVA or factorial designs were used.*

Specify type of analysis: ☐ Whole brain ☐ ROI-based ☐ Both

Statistic type for inference | *Specify voxel-wise or cluster-wise and report all relevant parameters for cluster-wise methods.*

(See Eklund et al. 2016)

Correction | *Describe the type of correction and how it is obtained for multiple comparisons (e.g. FWE, FDR, permutation or Monte Carlo).*

Models & analysis

n/a	Involved in the study
☐	☐ Functional and/or effective connectivity
☐	☐ Graph analysis
☐	☐ Multivariate modeling or predictive analysis

Functional and/or effective connectivity | *Report the measures of dependence used and the model details (e.g. Pearson correlation, partial correlation, mutual information).*

Graph analysis | *Report the dependent variable and connectivity measure, specifying weighted graph or binarized graph, subject- or group-level, and the global and/or node summaries used (e.g. clustering coefficient, efficiency, etc.).*

Multivariate modeling and predictive analysis | *Specify independent variables, features extraction and dimension reduction, model, training and evaluation metrics.*
